# Supplementary figures and images for: Investigation of the effects of estrogen on skeletal gene expression during zebrafish larval head development
Source: PeerJ. 2016 Mar 31;4:e1878. doi: 10.7717/peerj.1878 (PMC4824909; doi:10.7717/peerj.1878)

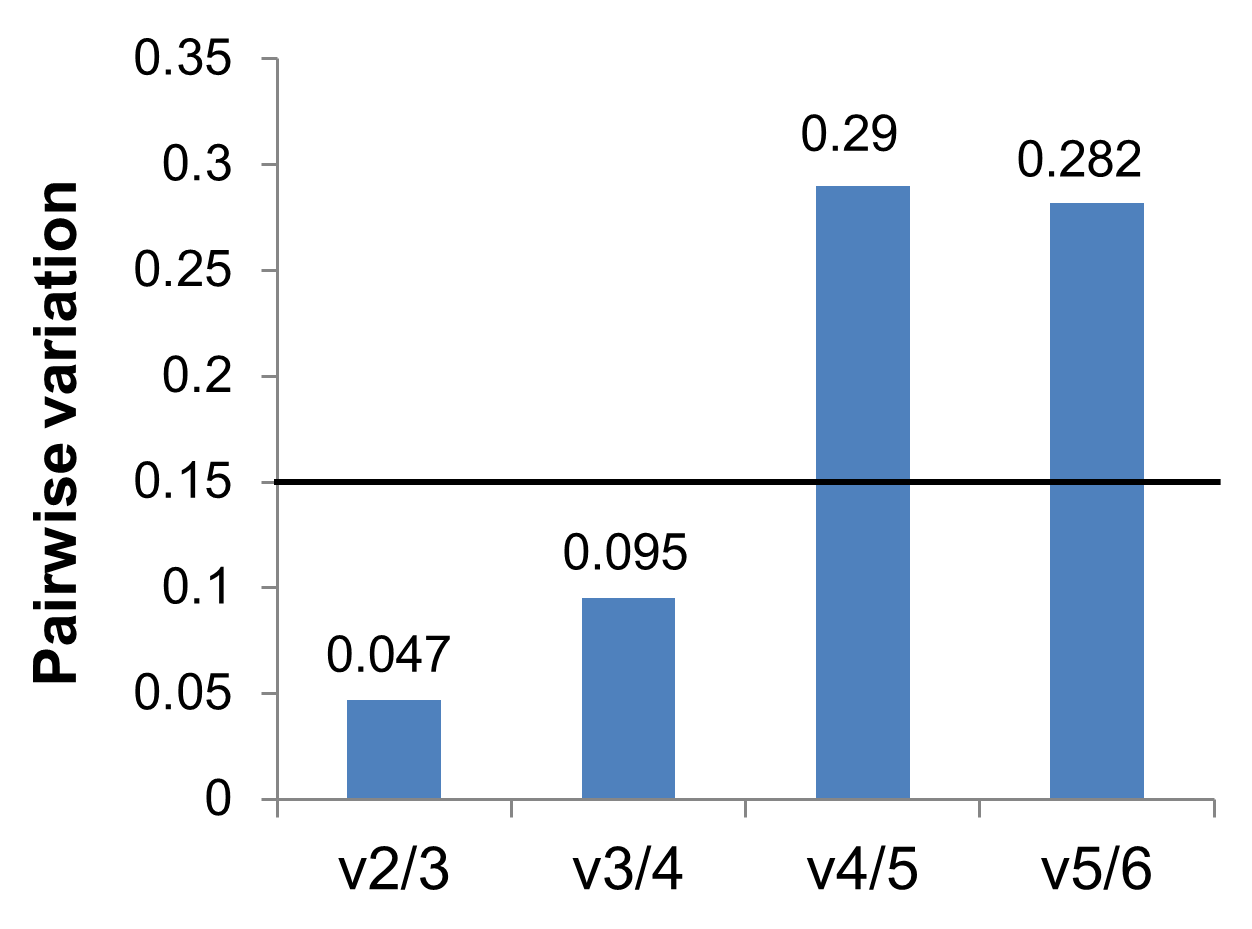

Supplement: Figure S1 — The algorithm used in geNorm software determines the optimal number of reference genes in developing heads of zebrafish larvae for all the treatments together (a control and two E2 treated groups). Average pairwise variations (Vn∕n+1) were calculated using the genes ranked according to geNorm. The recommended cut-off value of 0.15 is shown by a black line. Below this line the benefit of using an extra reference gene is limited. [file peerj-04-1878-s001.png]

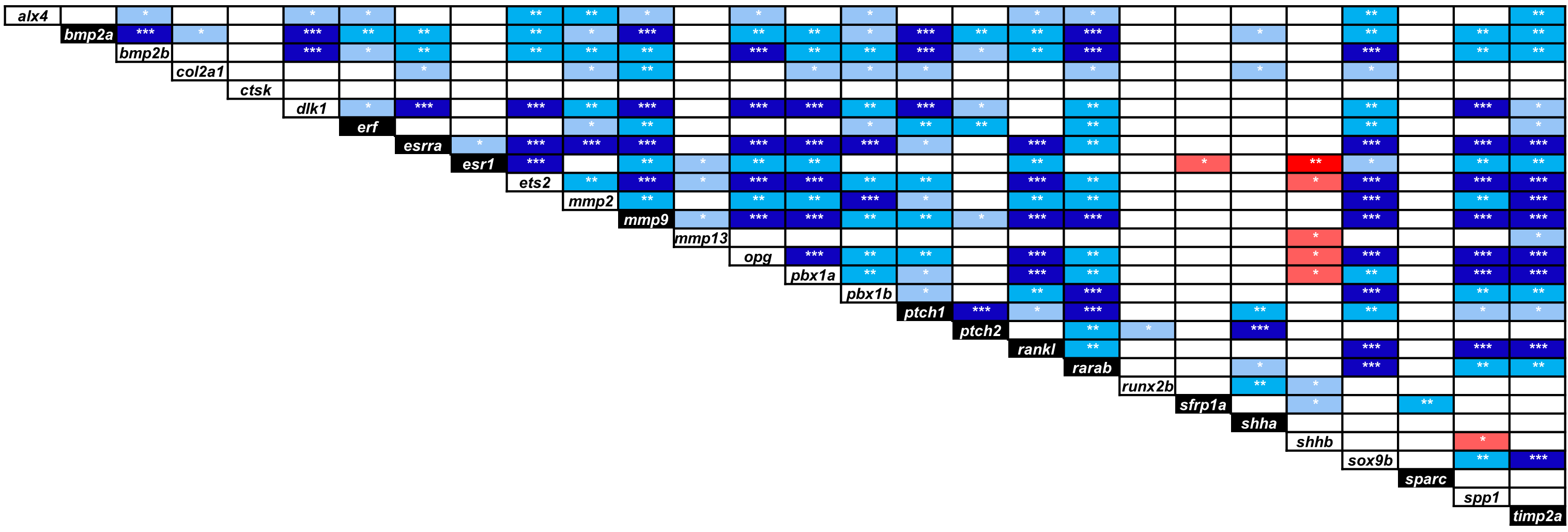

Supplement: Figure S2 — Pearson correlation coefficient (r) was used to assess the pairwise expression similarity between the candidate target genes. Blue shading represents positive and red shading represents negative expression correlation and the colour gradients indicate correlation coefficients (r) above critical values (2-tail; df = 13). P-values of <0.05, 0.01 and 0.001 are indicated by one, two and three asterisks, respectively. The genes showing differential expression between the treatment groups in at least three larval stages are represented in black boxes. [file peerj-04-1878-s002.png]

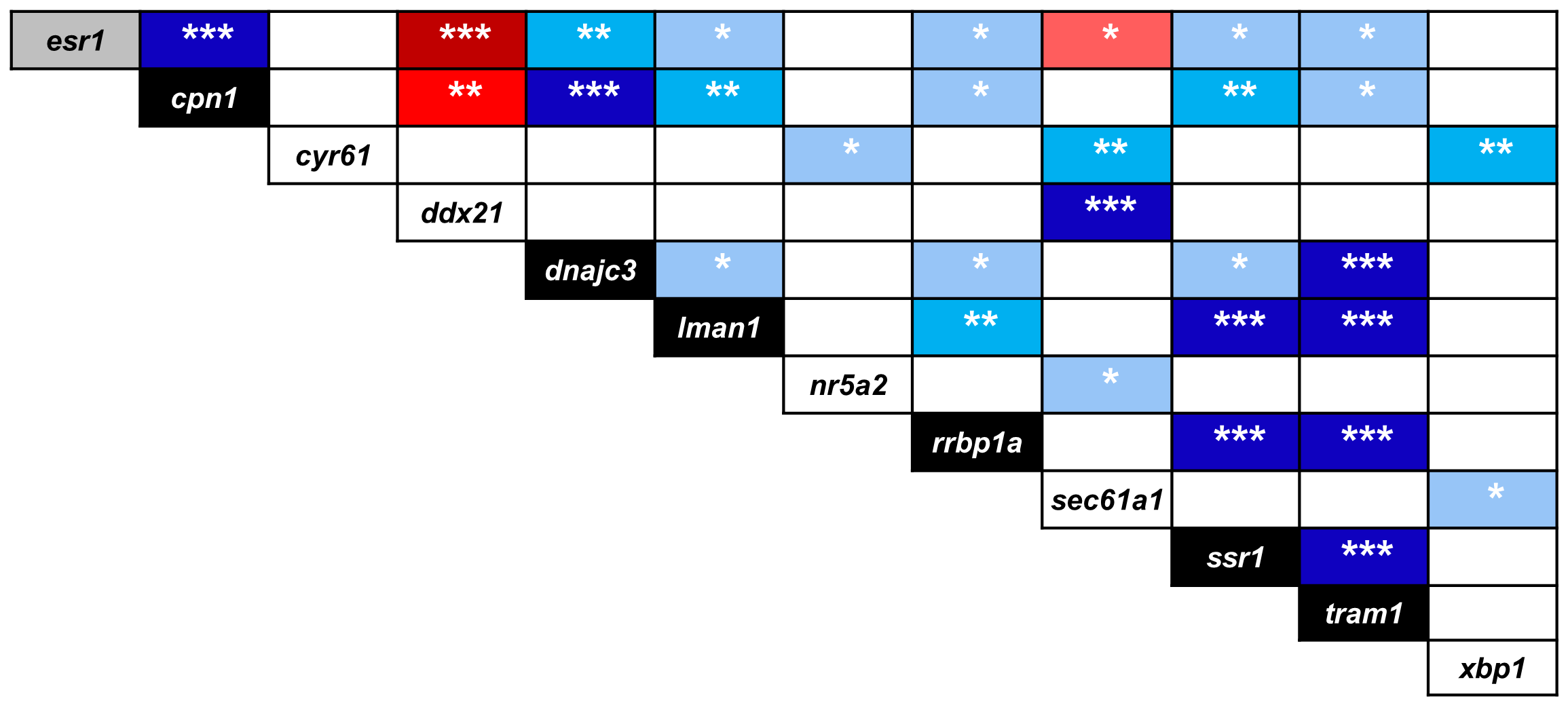

Supplement: Figure S3 — Pearson correlation coefficient (r) was used to assess the pairwise expression similarity between the candidate target genes. Blue shading represents positive and red shading represents negative expression correlation and the colour gradients indicate correlation coefficients (r) above critical values (2-tail; df = 13). P-values of <0.05, 0.01 and 0.001 are indicated by one, two and three asterisks, respectively. The genes showing differential expression between the treatment groups in at least three larval stages are represented in black boxes (except esr1 gene). [file peerj-04-1878-s003.png]
